# The meaning and importance of dignified care: findings from a survey of health and social care professionals

**DOI:** 10.1186/1471-2318-13-28

**Published:** 2013-03-22

**Authors:** Deborah Cairns, Veronika Williams, Christina Victor, Sally Richards, Andreé Le May, Wendy Martin, David Oliver

**Affiliations:** 1 School of Health Sciences and Social Care, Brunel University, Mary Seacole Building, Uxbridge UB8 3PH, UK; 2 Department of Primary Care Health Sciences, University of Oxford, Primary Care Clinical Trials Unit, Oxford, UK; 3 Department of Psychology, Oxford Brookes University, Oxford, UK; 4 University of Southampton, Southampton, UK; 5 City University School of Community and Health Sciences, Oxford, UK

**Keywords:** Dignity, Health care professionals, Social care professionals, Nursing, Older people, Ageing, Care, Hands on care

## Abstract

**Background:**

There are well established national and local policies championing the need to provide dignity in care for older people. We have evidence as to what older people and their relatives understand by the term ‘dignified care’ but less insight into the perspectives of staff regarding their understanding of this key policy objective.

**Methods:**

A survey of health and social care professionals across four NHS Trusts in England to investigate how dignified care for older people is understood and delivered. We received 192 questionnaires of the 650 distributed.

**Results:**

Health and social care professionals described the meaning of dignified care in terms of their relationships with patients: ‘respect’ (47%), ‘being treated as an individual’ (40%), ‘being involved in decision making’ (26%) and ‘privacy’ (24%). ‘Being treated as an individual’ and ‘maintaining privacy’ were ranked as the most important components of dignified care. Physical caring tasks such as ‘helping with washing, dressing and feeding’ were rarely described as being part of dignified care and attributed much less importance than the relational components.

**Conclusion:**

Dignity in care is a concept with multiple meanings. Older people and their relatives focus upon the importance of providing physical care when describing what this means to them. Our participants focussed upon the relational aspects of care delivery rather than care itself. Proactive measures are therefore required to ensure that the physical aspects of care are met for all older people receiving care in NHS trusts.

## Background

In the United Kingdom (UK), despite the range of policies and targets focused specifically upon delivering dignity in care for older people [1-3], there is substantial evidence that this is still being compromised [4-13]. The Parliamentary and Health Service Ombudsman report [7], for example, details 10 cases of elderly patients who died after being admitted to NHS hospitals but who did not receive the most basic standards of care such that they were left without food or water, were soaked in urine or lying in faeces and left on the floor after falling. In 2011 one in five hospitals inspected by the Care Quality Commission also exhibited ‘*basic failings*’ on dignity and nutrition [13]. The NHS Operating framework for 2012–2013 [14] prioritises the care of older people stating ‘*some parts of the NHS are failing to provide elderly and vulnerable patients with dignified and compassionate care or to offer good standards in areas such as nutrition*, *continence and communication*’ (p.2).

Dignity remains, however, a complex concept subject to a range of different interpretations [15]. Whilst the examples noted above operationalise dignity in terms of the delivery of fundamental nursing care, the Royal College of Nursing [5] definition emphasises core nursing values of respect and autonomy rather than care delivery:

“Dignity is concerned with how people feel, think and behave in relation to the worth or value of themselves and others. To treat someone with dignity is to treat them as being of worth, in a way that is respectful of them as valued individuals”

Similarly the more recent Social Care Institute for Excellence [16] focuses upon the relational aspects of care delivery rather than with delivery of care per se:

“Dignity is at the heart of personalisation. Dignity means treating people who need care as individuals and enabling them to maintain the maximum possible level of independence, choice and control over their own lives. It means that professionals should support people with the respect they would want for themselves or a member of their family”

Whilst older people cite respect, communication, privacy and being treated as an individual as important aspects of dignified care they emphasise the basic and vital aspects of care such as eating, nutrition, personal hygiene and toileting [11,17-22]. Less visible in the research literature is the professional perspective on delivering dignity in care and, more specifically, the educational, cultural and organisational factors which enable or hinder its delivery. This is a significant omission as it is the attitudes, skills and behaviour of frontline staff via the development of organisational culture, policies and practice which is critical to the tangible delivery of policy imperatives [23].

Hall and Høy [24] carried out one of the few studies exploring the professional perspective and more specifically, 29 Danish nurses’ experiences of caring for older hospital patients. Helping patients regain their dignity was considered to be of central importance to nurses. Participants reported that dignity was a value that had to do with integrity, respect and worthiness; something the older patients were in risk of losing when being hospitalised. A similar study was carried out to determine health and social care professionals’ views of various aspects of dignity and older people [25]. A total of 85 focus groups were carried out involving 424 professionals in six European countries. Participants’ views of what constitutes dignified care were highly consistent: dignified care promotes autonomy, independence, engenders respect, maintains individual identity, encourages involvement, adopts effective communication practices and is person-centred and holistic. However, the RCN [4] survey of the challenges faced by nurses in delivering dignified care did not seek their understanding, conceptualisation or definitions of dignity. Overall, while the limited research that has looked at the professional perspective is important it lacks the organisational perspective which our study addresses.

If dignified care for older people is to be implemented successfully, we need to fully understand both the patients’ and health care professionals’ understanding of dignity in order to develop appropriate and relevant policies and procedures to avoid the breaches of dignity in care of older people.

This paper reports findings on two key research questions from a survey of health and social care professionals which forms part of a larger case study (survey, interviews and focus groups) investigating how dignified care for older people is understood and delivered by health and social care professionals and; how organisational structures and policies can promote and facilitate, or hinder, the delivery of dignified care. The research questions that form the specific focus of this study are as follows:-

What does dignified care mean for health and social care professionals?

What are the most important aspects of dignified care as perceived by health and social care professionals?

## Methods

### Dignity questionnaire

We developed a self-completion questionnaire (Additional file 1) consisting of 22 questions, exploring health and social care staff perspectives and experiences of dignified care, which was modelled on the instrument developed by the Royal College of Nursing report [5] and informed by research which had taken place since the completion of that survey [26]. Overall 50% of our instrument was based on the RCN survey providing both a comparative context for our study. Both closed and open-ended questions were used to gain an insight into the experiences and perspectives of health and social care professionals. In addition participants were asked to provide standard demographic data (gender, age, ethnicity and job role).

Face validity for our measure was evaluated during the pilot testing of the questionnaire when fifteen health professionals were invited to provide feedback on the content of the instrument including: wording, layout, length, questions asked and also if there were any important questions missing. Eleven health professionals provided feedback and the questionnaire was refined accordingly by the research team. Only minor changes were made including font size and layout. No new items were identified for inclusion providing support for the face validity of our measure.

Data were collected for 12 months between June 2011 and June 2012. Ethical approval for the study was obtained (REC ref number: 10/H0711/49) from both Brunel University and the UK National Research Ethics Service (NRES).

### Participants and procedure

Our participants were drawn from four NHS Trusts (2 acute trusts, one mental health trust and one primary care trust) in England and who provided care for older people. Gatekeepers were identified within each Trust to assist with the recruitment of survey participants. Packs containing a letter of invitation to take part in the study, an information leaflet, a questionnaire and a stamped addressed envelope to return the questionnaire were provided to professionals who met the inclusion criteria (worked within one of the recruitment settings; were a health or social care worker; were over 18 years and; able to give informed consent). The main researcher (DC) visited all wards in both acute trusts where older people were cared for in some capacity and spoke with staff before leaving the packs. Additional questionnaires were left with ward managers and placed in staff rooms. For the mental health trust, packs of questionnaires were delivered by post to the main contact who then administered these to appropriate staff members. At the request of the primary care trust, an online version of the questionnaire was also developed for professionals to complete as a preferred method instead of hard copies. All trusts were sent the online questionnaire to encourage further participation and the acute and mental health trusts were visited on several occasions by members of the research team to ensure staff were aware of the project.

### Analysis

Data were cleaned and analysed using the Statistical Package for the Social Sciences (SPSS) version 18 and analysed using descriptive statistics in order to elucidate professionals’ understandings of the meaning and importance of dignified care. For the qualitative data within the survey a content analysis was used which is appropriate for a descriptive approach [27]. Content analysis determined the presence of certain words or codes within the text provided by participants. These codes were intended to have a wide scope to allow for variation within each category. The coding template continued to evolve as new information was collected. Once coding had been completed by the first author, DC, authors CV and VW carried out a separate analysis of a sub-set of the qualitative data to enable group validation of the emerging themes.

## Results

A total of 650 hard copies of questionnaires were administered across 3 of the 4 NHS trusts, of which 161 copies were returned (25% response rate). All 4 trusts received an email invitation to take part in the survey with a link to the online questionnaire. It is unknown how many professionals received this email as this was administered via administrative staff within each trust. A total of 31 questionnaires were completed online giving a total of 192 completed questionnaires.

### Profile of respondents

The majority of participants were female (86%) and the majority (82%) were aged between 25 – 54 years. The ethnicity of respondents was diverse but the majority 134 (70%) described themselves as White British. Participants were asked to select their job role from a list of options: most (n = 61) selected the category ‘staff nurse’. Participants who selected the category ‘other’ (n = 58) were asked to provide additional details about their job role: 45 reported that they had a nursing background such as sister, matron, ward sister, ward manager, junior sister, research nurse and specialist cancer nurse. The remaining 13 participants included 4 psychologists and one each of a diverse range of roles (for example, occupational therapy assistant; radiographer, podiatrist, psychiatrist,, consultant practitioner, physiotherapy assistant and social work assistant). In total, 109 (57%) of the respondents had a nursing background (see Table 1 for full profile of respondents).

**Table 1:** Profile of respondents

**Gender**	**n**	**%**
Male	27	14
female	165	86
**Age**	**n**	**%**
under 25	19	10
25-34	53	28
35-44	44	23
45-54	60	31
55+	16	8
**Ethnic Background**	**n**	**%**
White British	134	70
White Irish	6	3
Other white	15	8
White and Black Caribbean	1	.5
White and Asian	4	2
Other mixed	7	4
Indian	3	1
Pakistani	1	.5
Other Asian	10	5
Caribbean	3	2
African	6	3
Other Black	2	1
**Job Role**	**n**	**%**
Health care assistant	16	8
Staff nurse	61	32
Occupational Therapist	26	13
Physiotherapist	9	5
Social Worker	4	2
Medical doctor	5	3
Manager	13	7
Other	58	30

### The meaning of dignified care

Participants were asked to describe in their own words what dignified care meant to them and 177 (92%) provided a definition. Our content analysis identified 16 themes of which relational aspects of professional’s roles such as ‘respect’ and ‘being treated as an individual’ were the two most frequently cited; definitions being described by 83 (42%) and 70 (36%) of participants (see Table 2). Being involved in decision making, privacy and treating patients as you would wish to be treated were all cited by at least 15% of our participants. This table is informative for indicating what aspects of professional’s roles are not considered to define dignified care. The lack of prominence of definitions of dignity relating to the actual provision of direct care is striking.

**Table 2:** The meaning of dignified care

**Meaning of dignified care**	**n**	**Illustrative Quote (participant ID in brackets)**
Respect	83	*“dignified care means treating patients with respect”* (P16)
To be treated as an individual	70	*“ensuring the patients individual needs are taken into account during their “care” or in the planning of their “care””(*P6)
Involved in decision making	46	*“involving them in their treatment and discharge plans”* (P46)
Privacy	43	*“Ensuring privacy at all times”* (P2)
Treat as you/your family wish to be treated	35	*“To treat patients and families as you and you’re family would like to be treated”* (P27)
Care and support	27	*“providing care and support”* (P17)
Safe environment	13	*“feel safe in their environment”* (P104)
Listened to	12	*“being listened to”* (P106)
Needs are met	9	*“listening to their needs, wishes and respecting them”* (P162)
High standard of care	6	*“Ensuring you have time to deliver a high standard of care”* (P136)
Communication	5	*“Communicating to them and working with them”* (P151)
Basics in care	4	*“this identifies personal hygiene, eating and nutrition”* (P184)
Empathy	3	*“having the ability to empathize and understand the individuals predicament”* (P101)
Independence	2	*“ensuring they maintain their independence”* (P137)
Balance in care	2	*“balancing the clinical needs of a patient with their need for respect, time, security and involvement”* (P156)
Looking past mental health	1	*“Looking past the person (i.e. if they have dementia/aggressive/confused)”* (P141)

### The important aspects of dignified care

Respondents were also asked to rank in order of importance the 8 dimensions of dignified care. Mean scores (see Table 3) highlighted that ‘*treating a patient as an individual’* was considered the most important aspect of dignified care (ranked as 1) followed by ‘*maintaining privacy when providing care at all times and in all places*’ while the physical tasks such as ‘*providing adequate help with personal care*’ and ‘*helping patients at meal times’* were attributed much less importance.

**Table 3:** The most important aspects of dignified care

**Please rank in order how important these are to you (1 = most important, 8 = least important)**	**Mean**	**Order of importance**
Treating a patient as an individual	2.41	1
Maintaining privacy when providing care at all times and in all places	2.72	2
Responding promptly and professionally when patients ask for help	3.67	3
Having time to talk and actively listen to patients	4.18	4
Providing adequate help with personal care (e.g. washing, clothing, toileting)	4.84	5
Addressing patients as they have asked to be addressed	5.31	6
Helping patients at meal times	5.57	7
Obtaining consent from patients for sharing information	7.22	8

## Discussion

Our paper presents the perspectives of health and social care staff on the meaning and important elements of dignified care. ‘*Respect’, ‘being treated as an individual*’, ‘*being involved in decision making*’ and ‘*privacy*’; were the most frequently cited definitions and these resonate with dignity guidelines, protocols and definitions that are embedded in national/local policies and which mesh with the expressed views of older people [5,16,19-22]. However older people and various national reports also emphasise the importance of direct ‘hands on’ aspects of care including eating, nutrition, personal hygiene and toileting as an important component of dignified care [18,21] yet these dimensions of dignity were reported by only four participants in the current study (See Table 2). Similarly when professionals were asked what the most important aspects of dignified care were, they focused upon the relational aspects of care rather than the direct ‘hands on’: ‘*treating a patient as an individual’* as the most important aspect followed by ‘*maintaining privacy when providing care at all times and in all places*’; while the direct ‘hands on’ tasks such as ‘*providing adequate help with personal care*’ and ‘*helping patients at meal times’* were ranked 5th and 7th out of 8.

The staff in our study clearly conceptualised dignity as an approach to their role focussing upon ideas of respect, individuality and patient involvement; findings that resonate with previous studies looking at the professional perspective [24,25]. However unlike patients, few of our participants considered the direct ‘hands on’ aspects of care provision such as feeding and toileting as defining dignified care. One explanation for this disjuncture in definitions between staff could be the way that policies and debates about dignity are framed being concerned with attitude about how care should be delivered rather than how care is delivered. We may hypothesise that policy makers and practitioners are ‘taking for granted’ the implicit delivery of care embedded within their roles and see dignity as being concerned with how care is delivered. Alternatively the separation of tasks amongst health professionals may explain why professionals in our survey focussed upon the relational aspects of care. Divisions of responsibility for patient care between different groups of health and social care staff are a feature of the NHS but this may have important consequences for dignified care [6]. Tadd et al. [26] suggests that this may result in a lack of accountability for overall care whilst a recent, November 2011, meeting organised by the Nursing Standard, as part of their Care Campaign with the Patients Association, to discuss the neglect of essential tasks such as assistance with washing, toileting and feeding suggested this was a consequence of health professionals taking on more complex and specialist tasks [28]. It is plausible to suggest that direct ‘hands on’ or fundamental aspects of care are being neglected in staff definitions of dignity because of the specialisation and separation of roles and the emphasis in policy documents on how care is delivered (with the implicit assumption that essential physical care is being delivered)

### Limitations

Our findings come with a series of caveats. Whilst we have a large absolute sample this represents about a third of the total study population and it is not clear if our participants represent those most (or least) engaged with the dignity agenda. However, similar findings were reported by the RCN [5] who attracted only a small fraction (n = 2,048) of their total Membership (n = 600,000). In terms of gender and ethnicity, our sample reflects the general NHS health care professional population [5]. We also acknowledge that our paper has focussed upon understanding the meaning of dignity from an empirical and policy perspective rather than as philosophical or psychological concepts. We intend to deal with this aspect of our study in later publications.

## Conclusion

Our study highlights the disjuncture between staff and patient expectations as to what constitutes dignified care. Furthermore the lack of importance attributed to the vital aspects of care suggests that policies around providing dignified care are being interpreted as an approach towards care and not with direct care provision. We suggest that this limited interpretation of dignity may be one factor contributing to the continued neglect of older people in acute settings. Policy makers, NHS organisations, managers, medical doctors, nurses and health and social care professionals more generally, equally have a duty of care to address the vital aspects of dignified care. In order to support and encourage health and social care professionals, proactive measures are required. For example, ‘intentional patient rounding’ has been suggested where a nurse carries out ward visits every couple of hours and asks patients if they have everything they need [28]. Thus, aspects of care including eating, nutrition, personal hygiene and toileting would be monitored on a regular basis to ensure that the direct ‘hands on’ aspects of care are met for all older people receiving care in NHS trusts. Phase II (in-depth interview) of this study will explore further the meaning and importance of dignified care to ascertain why the direct ‘hands on’ aspects of care are accorded less importance.

### Ethics approval

Ethical approval for the study was obtained (REC ref number: 10/H0711/49) from both Brunel University and the UK National Research Ethics Service (NRES).

## Competing interests

The authors have no competing interests.

## Authors’ contributions

DC carried out the data collection and analysis and drafted the manuscript. CV is the Chief Investigator on the project and made substantial contributions to the conception and design of the study and helped to draft the manuscript. VW participated in the design of the study and reviewed the manuscript. SR participated in the design of the study and reviewed the manuscript. AM participated in the design of the study and reviewed the manuscript. WM participated in the design of the study and reviewed the manuscript. DO made substantial contributions to the conception and design of the study and reviewed the manuscript. All authors read and approved the final manuscript.

## Authors’ information

Co-authors: Veronika Williams, Christina Victor, Sally Richards, Andreé Le May, Wendy Martin and David Oliver.

## Funding

This research was supported by the Dunhill Medical Trust [grant number: R93/1108].

## Pre-publication history

The pre-publication history for this paper can be accessed here:

http://www.biomedcentral.com/1471-2318/13/28/prepub

## Supplementary Material

Additional file 1 Dignity survey. Click here for file
