# Supplementary material for: The meaning and importance of dignified care: findings from a survey of health and social care professionals
Source: BMC Geriatr. 2013 Mar 22;13:28. doi: 10.1186/1471-2318-13-28 (PMC3614439; doi:10.1186/1471-2318-13-28)
Supplement: Additional file 1 — Dignity survey. [file 1471-2318-13-28-S1.pdf]

Bridging the gap between policy and practice – Dignity in Care for Older People

STAFF SURVEY

We are a team of researchers from Brunel University (Prof Christina Victor, Dr Deborah Cairns, Dr Veronika Williams and Dr Wendy Martin), Oxford Brookes University (Dr Sally Richards), Formerly the University of Southampton (Prof Andree Le May) and City University London (Prof David Oliver).

Much research has been carried out to explore patients' views on dignified care but we know very little about the views and experiences of staff working with older people (older people can range from early sixties to the oldest patients within your care). We are therefore undertaking a research project aiming to explore the perspectives and experiences of frontline care staff on dignified care. We hope this will help us to better understand how staff think about dignity in care and how the organisational structure of care environments can be improved to facilitate the delivery of dignity in care.

We would be delighted to hear your views on this important subject and invite you to take part in this survey. Please answer your questions by ticking the appropriate box. Please do not provide personal details that may identify you or the trust where you work. As this survey will be anonymous, we will not be able to withdraw your completed questionnaire once you have sent it to us.

Many thanks for your time in completing this survey.

1. **How much of your work time is spent working with older people? (both direct care and indirect care, eg administrative work)**

   Less than 25% ☐
   26-49% ☐
   50-75% ☐
   76- 100% ☐

2. **Please rate each of these aspects of care according to how important they are for the wellbeing of older people in hospital (1= most important, 10 = least important). Please circle your answer.**

**Undertaking clinical treatments** (e.g. taking clinical observations, clinical interventions, administration of medicines)	most important … least important<br>1 2 3 4 5 6 7 8 9 10
**Ensuring that all documentation is up to date**	most important … least important<br>1 2 3 4 5 6 7 8 9 10
**Having time to talk and actively listen to patients**	most important … least important<br>1 2 3 4 5 6 7 8 9 10
**Ensuring that patients receive care tailored to their individual needs**	most important … least important<br>1 2 3 4 5 6 7 8 9 10
**Making sure patients have been provided with meals on time and helped with eating and drinking as appropriate**	most important … least important<br>1 2 3 4 5 6 7 8 9 10
**Making sure patients have been given the opportunity to receive help with washing, dressing and toileting, if required**	most important … least important<br>1 2 3 4 5 6 7 8 9 10

**Ensuring that patients are safe**	most important … least important<br>1 2 3 4 5 6 7 8 9 10
**Ensuring that patients are comfortable**	most important … least important<br>1 2 3 4 5 6 7 8 9 10
**Ensuring confidentiality and privacy of patient data**	most important … least important<br>1 2 3 4 5 6 7 8 9 10
**Good communication and collaboration within the multi-disciplinary team**	most important … least important<br>1 2 3 4 5 6 7 8 9 10
**Making sure that patients are involved in discharge planning**	most important … least important<br>1 2 3 4 5 6 7 8 9 10
**Making sure that patients are kept fully informed and up to date about plans for discharge**	most important … least important<br>1 2 3 4 5 6 7 8 9 10

3. Please describe what dignified care means to you:

....

....

....

....

....

4. **Which of these aspects of care do you consider to form part of dignified care for older people? (please tick all that apply)**

Ensuring confidentiality and privacy ☐

Having time to talk and actively listen to patients ☐

Respecting patients needs and views on their care ☐

Providing clinical care on time and to a high standard ☐

Providing adequate help and suitable environments for meal times, washing and dressing ☐

Providing same sex care environments ☐

Ensuring patients are given appropriate clothing whilst in hospital ☐

Ensuring that patients understand their treatment, have provided informed consent and have been given choices with regard to their care ☐

Are there any other aspects of care that you consider part of dignified care for older people? Please specify.

....

....

....

....

....

5. **Do you think that the features of dignified care for older people are different from other groups?**

Yes ☐
No ☐
Don't know ☐

If Yes-in what ways is it different? Please specify

..

..

..

..

6. **In your view how important is the provision of dignified care for older people in:**

	**Very important**	**Important**	**Unimportant**	**Don't know**
**Your organisation**				
**Your ward/work area**				
**Your work colleagues**				
**Yourself**				

7. **How often do you estimate that you are able to deliver dignified care on a daily basis?**

Never ☐
Sometimes ☐
About half of the time ☐
Most of the time ☐
All of the time ☐

8. Are there specific circumstances/factors that help you to provide dignified care?

Yes ☐
No ☐
Don’t know ☐

If Yes-what are these? Please specify

..

..

..

..

9. Are there specific circumstances/factors that prevent you from providing dignified care?

Yes ☐
No ☐
Dont know ☐

If Yes-what are these? Please specify

..

..

..

..

10. Please rank in order of importance how important these aspects of dignified care are to you (1= very important, 8= least important)

**Treating a patient as an individual**	
**Addressing patients as they (or their carers) have asked to be addressed**	
**Maintaining privacy when providing care at all times and in all places**	
**Having time to talk and actively listen to patients**	
**Responding promptly and professionally when patients ask for help**	
**Helping patients at meal times**	
**Obtaining consent from patients for sharing information**	
**Providing adequate help with personal care (eg washing, clothing, toileting)**	

11. How easy do you find it to deliver the following aspects of dignified care at your work place:

	Easy	Neither easy nor difficult	Difficult	Further comments
Maintaining privacy when providing personal care				
Providing help with meals				
Access to side rooms to talk to patients in privacy				
Providing individualised spaces/ furniture for patients (such as lockable side cupboards)				
Provision of a clean care environment				
Having time to talk and actively listen to patients when delivering care				
Providing adequate information to patients about their care				
Being able to involve patients in their care and decisions about their care				
Ability to respect the patient's personal needs and care preferences at all times				
Ability to promote patients autonomy and right to make independent choices				

12. Does your employer support you in delivering dignified care in the following ways:

	Yes	Somewhat	No	Don't know
**Our work place philosophy specifically mentions dignity in care**				
**The importance of providing dignified care is included in new staff induction**				
**We have internal development events that include training on dignified care**				
**We have a good skill mix**				
**We have good staffing levels**				
**I am able to discuss difficult issues of dignity with my colleagues**				
**I am able to include dignity in care when teaching/ working with students/ new staff**				
**I feel able to report breaches of dignity in care in confidence to my manager/ employer (whistle blowing)**				

13. Would you like to tell us more about any specific issues with regard to how your employer/ organisation either supports/does not support you in delivering dignified care? Please give examples

....

....

....

....

14. How would you rate the standard of provision of dignified care to patients by:-

	Excellent	Good	Fair	Poor
Your organisation				
Your ward/work area				
Your work colleagues				
Yourself				

15. Within your role as a care professional providing dignified care do you feel able to:

	Yes	No	if no please explain
Develop your understanding of dignified care (eg through training)			
Reflect on the care you provide			
Enhance dignity in care by being a role model to other staff			
Challenge attitudes and behaviours by others that diminish dignity in care			

16. Are there things about your organisation that:

	Yes	No	If Yes Please give details
Help you to deliver dignified care			
Prevent you from delivering dignified care			
Need to change to help you deliver dignified care			

17. Does your clinical area have a dignity champion?

Yes ☐
No ☐

If yes, have you had any contact with them?

Yes ☐
No ☐

18. Are there things about your physical working environment that:

	Yes	No	If Yes Please give details
Help you to deliver dignified care			
Prevent you from delivering dignified care			
Need to change to help you deliver dignified care			

19. Are there specific procedures and/or activities that you feel make older patients vulnerable to a loss of dignity?

Yes ☐
No ☐
Don't know ☐

If Yes-what are these? Please specify

....

....

20. Have you had experience of an elderly friend or relative receiving in-patient hospital care?

Yes ☐
No ☐

If Yes - what was important to you with regard to maintaining their dignity?

....

....

If Yes - did you have any concerns about their experience of dignified care?

Yes ☐
No ☐

If Yes - what were these? Please list these for us

....

....

....

21. Which of these would help you to maintain and improve your ability to provide dignified care?

Please tick the three most important aspects only and rate them 1 – 3 (1= most important)

**Education**	☐
**Peer support**	☐
**Support from your managers/ organisation**	☐
**Better staffing**	☐
**Less work pressure**	☐
**More time**	☐
**Better work environment (e.g. equipment, cleanliness, space)**	☐
**Integration of dignity into work philosophy**	☐

Are there any other factors that you think are important?

Please specify

....

....

....

22. Finally could you provide some details about yourself.

a) Have you ever received training on dignity in care?

Yes ☐
No ☐

If Yes where was this? (Please tick all that apply)

Initial training (in classroom) ☐
Initial training (in practice) ☐
Continuous professional development ☐
During employment ☐
None of the above ☐
Other please specify........ ☐

…………………………………………………………………………………………..

b) Which age group do you belong to?

Under 25 ☐

25-34 ☐

35-44 ☐

45-54 ☐

55+ ☐

c) Gender

Male ☐

Female ☐

d) Ethnic background:

White British ☐

White Irish ☐

Other White ☐

White and Black Caribbean ☐

White and Black African ☐

White and Asian ☐

Other mixed ☐

Indian ☐

Pakistani ☐

Bangladeshi ☐

Other Asian ☐

Caribbean ☐

African ☐

Other Black ☐

e) What is your role?

Health care assistant ☐

Staff nurse ☐

Lecturer in practice ☐

Occupational therapist ☐

Physiotherapist ☐

Social worker ☐

Medical doctor ☐

Manager ☐

Other-please specify ………………..

Add additional information about your job role if you wish to do so.

...

...

f) Where do you work?

Acute Hospital Trust ☐

A&E ☐

General medical ☐

General surgical ☐

Orthopaedics ☐

Elderly Care ☐

Primary Care Trust ☐

Mental Health Trust ☐

Other-please specify ………………..

g) Do you believe that you are treated with dignity/valued at work by:

	Yes	no	Don't know
**Your organisation**			
**Your work colleagues**			
**Patients**			
**Families/carers**			

Is there anything else about providing dignified care for older people that you would like to tell us?

....

....

....

....

....

....

....

THANK YOU FOR COMPLETING OUR SURVEY

Please return it in the stamped addressed envelope provided

If you would like to take part in the focus groups and/or interviews please print your name and a contact phone number on the separate sheet enclosed and return this with your survey.
